# Development of a Multivalent Kunjin Virus Reporter Virus-Like Particle System Inducing Seroconversion for Ebola and West Nile Virus Proteins in Mice

**DOI:** 10.3390/microorganisms8121890

**Published:** 2020-11-29

**Authors:** Pham-Tue-Hung Tran, Naveed Asghar, Urban Höglund, Olivia Larsson, Lars Haag, Ali Mirazimi, Magnus Johansson, Wessam Melik

**Affiliations:** 1 School of Medical Science, Inflammatory Response and Infection Susceptibility Centre (iRiSC), Örebro University, 703 62 Örebro, Sweden; hung.tran@oru.se (P.-T.-H.T.); naveed.asghar@oru.se (N.A.); magnus.johansson@oru.se (M.J.); 2 Adlego Biomedical AB, P.O. Box 42, 751 03 Uppsala, Sweden; urban.hoglund@adlego.se (U.H.); Olivia.larsson@adlego.se (O.L.); 3 EM Unit (EMil), Department of Laboratory Medicine, Karolinska Institute, 171 77 Solna, Sweden; lars.haag@ki.se; 4 Division of Clinical Microbiology, Department of Laboratory Medicine, Karolinska Institutet, 141 52 Huddinge, Sweden; ali.mirazimi@ki.se; 5 National Veterinary Institute, 751 89 Uppsala, Sweden

**Keywords:** reporter virus-like particles (RVPs), replicons, Kunjin virus, Ebola virus, glycoprotein (GP), matrix protein (VP40), packaging system, stable cell line, seroconversion, vaccines

## Abstract

Kunjin virus (KUNV) is an attenuated strain of the severe neurotropic West Nile virus (WNV). The virus has a single-strand positive-sense RNA genome that encodes a polyprotein. Following gene expression, the polyprotein is cleaved into structural proteins for viral packaging and nonstructural proteins for viral replication and expression. Removal of the structural genes generate subgenomic replicons that maintain replication capacity. Co-expression of these replicons with the viral structural genes produces reporter virus-like particles (RVPs) which infect cells in a single round. In this study, we aimed to develop a system to generate multivalent RVPs based on KUNV to elicit an immune response against different viruses. We selected the Ebola virus (EBOV) glycoprotein (GP) and the matrix protein (VP40) genes, as candidates to be delivered by KUNV RVPs. Initially, we enhanced the production of KUNV RVPs by generating a stable cell line expressing the KUNV packaging system comprising capsid, precursor membrane, and envelope. Transfection of the DNA-based KUNV replicon into this cell line resulted in an enhanced RVP production. The replicon was expressed in the stable cell line to produce the RVPs that allowed the delivery of EBOV GP and VP40 genes into other cells. Finally, we immunized BALB/cN mice with RVPs, resulting in seroconversion for EBOV GP, EBOV VP40, WNV nonstructural protein 1, and WNV E protein. Thus, our study shows that KUNV RVPs may function as a WNV vaccine candidate and RVPs can be used as a gene delivery system in the development of future EBOV vaccines.

## 1. Introduction

The genus *Flavivirus* (family *Flaviviridae*) includes important viruses that cause worldwide morbidity and mortality. The genus includes the mosquito-borne neurotropic West Nile virus (WNV) whose infection may lead to severe meningoencephalitis. Over the last two decades, the virus has caused more than 2000 fatal cases in America [1]. Contrarily, the Kunjin virus (KUNV) is a naturally attenuated WNV strain, which has caused a limited number of nonfatal cases since 1960 [2,3]. The difference in virulence of these two viruses is due to the high sensitivity of KUNV to the type I interferon response [4]. The KUNV nonstructural protein (NS) 5, in fact, cannot inhibit interferon-dependent JAK–STAT signaling as the WNV NS5 [5].

The flaviviruses are enveloped, icosahedral particles of approximately 50 nm in diameter. The particles encapsidate an 11 kb single-strand positive-sense RNA genome, which possesses a conserved 5′-RNA cap but lacks a 3′-polyadenylated tail. The genome, which also serves as viral mRNA, codes for a single polyprotein that is subsequently cleaved by viral and cellular proteases to generate three structural proteins: capsid (C), precursor membrane (prM), envelope (E) for viral packaging, and seven nonstructural proteins: NS1, NS2A, NS2B, NS3, NS4A, NS4B, and NS5. The polyprotein-coding gene is flanked by 5′- and 3′-untranslated regions (UTRs) containing sequences and structural motifs that are essential for a number of viral lifecycle processes, such as translation, replication, and assembly [6,7].

Substitutions of the structural genes in flaviviral genomes with reporter genes, such as luciferase or green fluorescent protein, lead to the generation of subgenomic replicons which maintain the capacity for viral replication. KUNV was the first flavivirus replicon system constructed and studied by Khromykh et al. [8]. Since then, several flavivirus replicon systems have been established [9,10,11,12] to study viral replication and specific functions of the NS proteins. Furthermore, co-expression of flavivirus replicons with the corresponding structural genes results in the packaging of replicon RNA, which generates reporter virus-like particles (RVPs) having potential for vaccine developments [13,14,15,16]. 

Ebola virus is an enveloped, single-stranded negative-sense RNA virus that belongs to the *Filoviridae* family. The *Ebolavirus* comprises six distinct species: *Zaire ebolavirus* (EBOV), *Bombali ebolavirus, Bundibugyo ebolavirus*, *Sudan ebolavirus*, *Taï Forest ebolavirus*, and *Reston ebolavirus* [17]. EBOV is considered to be the most dangerous species with the highest fatality rate of all the hemorrhagic fever diseases in West Africa [18]. The EBOV genome is approximately 19 kb in size and encodes the nucleoprotein (NP), polymerase cofactor (VP35), matrix protein (VP40), glycoprotein (GP), transcriptional activator (VP30), RNA complex-associated protein (VP24), and RNA-dependent RNA polymerase (L). 

The envelope surface glycoprotein GP that is approximately 150 kDa [19] is responsible for cell attachment, fusion, and cell entry. The role of the viral GP makes it an interesting antigenic target for Ebola vaccine candidates as it has good immunogenicity and can induce the generation of neutralizing antibodies. Thus, it is the potential candidate for use in the development of a vaccine against EBOV [20]. In addition, EBOV VP40 is a matrix protein located between nucleoproteins and the virus membrane. The protein is made up of 326 amino acids and is approximately 40 kDa [21]. The expression of VP40 can generate empty virus-like particles (VLPs) [22] that elicit immune responses [23,24,25,26]. 

In this study, we aimed to characterize the safety and the efficacy of KUNV RVPs to deliver genes of interest in infected cells. We explored the potential of using KUNV-based RVPs as a multivalent vaccine strategy. We generated KUNV RVPs that deliver EBOV GP or VP40 genes in infected cells. These RVPs were then administered to mice to examine their potential as vaccine candidates. The administration of RVPs into mice induced seroconversion, generating antibodies against EBOV GP, EBOV VP40, WNV NS1, and WNV E. The data in this study highlight a new strategy to develop multivalent EBOV and WNV vaccines. 

## 2. Materials and Methods 

### 2.1. Cell Culture

The Baby hamster kidney cell line (BHK-21) (ATCC) was maintained in Dulbecco’s modified Eagle’s medium (DMEM) (Gibco, New York, NY, USA) containing 1 g/L glucose (Gibco) supplemented with 10% heat-inactivated fetal bovine serum (Gibco), 100 U/mL penicillin–streptomycin (Gibco), and 1% nonessential amino acids (Gibco) at 37 °C in 5% CO_2_. 

### 2.2. Preparation of Gene Constructs

The KUNV C-prM-E sequence (accession number AY274504) was chemically synthesized and then cloned into a modified mammalian expression vector pCAGGS (Belgian Coordinated Collections of Microorganisms). To generate the KUNV C-prM-E-IRES-NeoR/KanR construct, the C-prM-E sequence was fused with an internal ribosome entry site (IRES) sequence and a neomycin/kanamycin resistance gene which confers G418 antibiotic resistance, followed by a polyadenylation signal (pA).

The KUNV replicon was constructed based on the KUNV sequence (accession number AY274504). The replicon sequences were chemically synthesized in two parts which were ligated to generate a DNA-based KUNV. Briefly, the replicon is driven by a Cytomegalovirus (CMV) promoter expressing an open reading frame (ORF) of KUNV where most of the structural cassette is replaced with firefly luciferase reporter gene (*Luc*). The foot-and-mouth disease virus autoprotease 2a sequence was fused in frame after *Luc* for post-translational cleavage release of the reporter. The ORF is flanked by the 5′-UTR and the 3′-UTR and a hepatitis delta virus antigenomic ribozyme sequence was inserted immediately downstream of the KUNV 3′-UTR, followed by the Simian virus 40 polyadenylation signal.

### 2.3. Establishment of a Stable Cell Line Expressing the KUNV Packaging System

The KUNV C-prM-E-IRES-NeoR/KanR construct was linearized before electroporation into BHK-21 cells using a Nucleofector system (Lonza, Basel, Switzerland). Two days after transfection, the cell culture was supplemented with 600 µg/mL G418 (Invivogen, Toulouse, France) to select for transfected cells. After 2 weeks, the transformants were established and were separated into single cells and seeded into multi-well plates before being grown into clones.

### 2.4. Antibodies 

For immunofluorescence and immunoblotting, the following antibodies were used: mouse monoclonal anti-flavivirus E (Abcam, Cambridge, UK), rabbit anti-EBOV GP (Genetex, Hsinchu City, Taiwan), rabbit anti-EBOV VP40 (Genetex), mouse monoclonal J2 anti-dsRNA (Scicons, Szirák, Hungary), mouse anti-GAPDH (Sigma, St. Louis, MO, USA), Alexa Fluor 594-conjugated anti-mouse goat antibody (Invitrogen), Alexa Fluor 488-conjugated anti-rabbit goat antibody (Invitrogen, Carlsbad, CA, USA), and HPR-conjugated anti-mouse goat antibody (Invitrogen).

### 2.5. Protein Electrophoresis and Immunoblotting

Cell lysates were prepared using RIPA buffer (Thermoscientific, Waltham, MA, USA) with protease inhibitors (Sigma) for 20 min at 4 °C before boiling in LDS sample buffer (Invitrogen). Proteins were separated on precast 4–12% polyacrylamide Bis-Tris gels (Invitrogen) in MES running buffer (Invitrogen) for 60 min at 130 V constant before being transferred to nitrocellulose membranes using the iBlot 2 Gel Transfer Device (Invitrogen). Proteins of interest were detected with the antibodies anti-flavivirus E (1:100), anti-EBOV GP (1:500), anti-EBOV VP40 (1:500), and anti-GAPDH (1:2000).

### 2.6. Immunofluorescence Labeling

Cells grown on coverslips were fixed by 4% paraformaldehyde (Scharlau, Barcelona, Spain) for 20 min. They were then permeabilized by 0.1% Triton X-100 (VWR) before blocking with 2% bovine serum albumin (Fitzgerald, MA, USA) and 2% goat serum (Invitrogen). Cells were labeled with the primary antibodies anti-flavivirus E (1:100), anti-EBOV GP (1:500), and anti-EBOV VP40 (1:500), for 1 h at 37 °C, followed by the secondary antibodies Alexa Fluor 594 (1:500) and Alexa Fluor 488 (1:1000). Images were captured using the confocal laser scanning microscopy SP8 (Leica, Wetzlar, Germany) and analyzed using ImageJ. 

### 2.7. RVP Infection and RVP Concentration Measurement

Supernatants from the RVP producing system were collected and filtered to remove detached cells. They were then used to infect confluent BHK-21 cells for 1 h at 37 °C in 5% CO_2_. Two days after infection, the cells were subjected to immunofluorescence labeling prior to microscopy to count the number of infected cells representing the number of RVPs in the supernatants.

### 2.8. Transmission Electron Microscopy (TEM)

We applied 3 µL of supernatants on glow-discharged carbon-coated formvar-stabilized 400 mesh copper grids (Ted Pella, Redding, CA, USA), followed by incubation for approximately 30 s. The grid was then washed with MilliQ water prior to negative staining using 2% uranyl acetate. Imaging was conducted using the HT7700 (Hitachi High-technologies, Tokyo, Japan) transmission electron microscope operated at 100 kV and equipped with a 2k × 2k Veleta charge-coupled device (CCD) camera (Olympus Soft Imaging System, Muenster, Germany).

Cells were fixed in 2.5% glutaraldehyde in 0.1 M phosphate buffer pH 7.4 at room temperature (RT) for 1 h, followed by storage at 4 °C. Following the primary fixation, the cells were rinsed with 0.1 M phosphate buffer and post-fixed in 2% osmium tetroxide in 0.1 M phosphate buffer, pH 7.4 at 4 °C for 2 h. The cells were then ethanol dehydrated stepwise, followed by stepwise acetone/LX-112 infiltration, and finally embedded in LX-112 (Ladd, Williston, VT, USA). Semi-ultrathin sections were prepared using the EM UC 7 (Leica). The ultrathin sections (approximately 60–80 nm) were contrasted with uranyl acetate followed by Reynolds’ lead citrate and examined in the HT7700 transmission electron microscope (Hitachi High-Tech) operated at 100 kV. Digital images were acquired using a 2k × 2k Veleta CCD camera (Olympus Soft Imaging Solutions GmbH, Münster, Germany). 

### 2.9. Luciferase Assay 

Cell lysates were assayed and measured for bioluminescence using the Dual-Luciferase Reporter Assay kit (Promega, Madison, WA, USA) and the Lumi-star Omega machine (BMG Labtech, Ortenberg, Germany), according to the manufacturer’s instructions. 

### 2.10. Quantitative Real-Time PCR (qPCR)

Total RNA was isolated from cell lysates or cell culture supernatants using the RNeasy Mini kit (Qiagen, Hilden, Germany) or the QIAamp Viral RNA Mini Kit (Qiagen), respectively. cDNA was synthesized from the isolated RNA using the High-Capacity cDNA Reverse Transcription kit (Applied Biosystems, Forster City, CA, USA) with a specific primer targeting the KUNV positive strand (5′- CGGCCTGACTTCCTCACTAAA-3′). qPCR was conducted using the QuantStudio 7 Flex Real-Time PCR System (Applied Biosystems) with the TaqMan Fast Advanced Master Mix (Applied Biosystems) and a Custom TaqMan Gene Expression Assay (Applied Biosystems) having the forward primer 5′-AGAGAAGCGGTTGAAGATCCAAAAT-3′, the reverse primer 5′-AGTATGACATTCTCCGCGTAAGTG-3′, and the probe FAM-5′- TCATCCACCATTTCCC-3′-NFQ. 

### 2.11. RVP Purification

The RVPs were purified through a 25% sucrose cushion using ultracentrifugation. In short, supernatants from the RVP production system were collected and filtered through a 0.2 µm filter to remove dead cells before supplementing with 10 mM HEPES (Gibco). Thereafter, 25 mL of the supernatants were loaded in 38 mL ultracentrifugation tubes (Beckman) containing 10 mL of 25% sucrose (Sigma) supplemented with 10 mM Tris-base (Sigma), 0.1 mM EDTA (Sigma), and 10 mM HEPES (Gibco) (pH 7.6). The centrifugations were performed at 150,000× *g* for 2 h at 4 °C using the swing rotator SW-32Ti in the Optima XPN-80 ultracentrifuge (Beckman, Fullerton, CA, USA). After centrifugation, both the supernatant and the sucrose were removed, and the RVPs were dissolved in 200 µL of DMEM supplemented with 10 mM HEPES (Gibco). 

### 2.12. Animal Immunizations 

All animal studies were conducted in accordance with ethics approval (4570-2019) by the Regional Animal Experimental Ethics Committee in Stockholm (North), Sweden. Six-week-old female BALB/cN mice (Charles River, Germany) were subcutaneously immunized three times at weeks 0, 2, and 5 with 200 µL of concentrated RVPs (approximately 10^6^ RVPs) carrying replicons expressing EBOV GP (6 mice), EBOV VP40 (6 mice), or luciferase as control 1 (6 mice). Another control group of three mice received 200 µL of phosphate-buffered saline (PBS) (Gibco). 

### 2.13. Sample Collection

Three weeks after the last RVP immunization, all mice were euthanized. The mice were anesthetized with isoflurane and blood samples were collected from their orbital plexus. The blood was incubated at RT for 30 min before centrifugation at 2000× *g* for 10 min at 20 °C. The sera were collected and stored at −80 °C until use.

### 2.14. Enzyme-Linked Immunosorbent Assays (ELISA)

To quantify the EBOV GP, EBOV VP40, WNV NS1, and WNV E antibodies in the sera, sera diluted 100× were assayed using ELISA kits (Alpha Diagnostic International, San Antonio, TX, USA), according to manufacturer’s instructions. The absorbance was read at 450 nm using the Cytation 3 Multi-Mode Reader (BioTek, Bad Friedrichshall, Germany). 

### 2.15. Statistics

Statistical differences between the means were determined using Student’s *t*-test, and *p* < 0.05 was considered to indicate a statistically significant difference in the comparison of two groups. Likewise, one-way ANOVA followed by the Bonferroni post hoc test was employed for comparing more than two groups. GraphPad Prism was used for performing all statistical analyses. The values are presented as the mean ± the standard error of the mean. 

## 3. Results

### 3.1. Establishment of the Stable Cell Line Expressing the KUNV Packaging System C-prM-E

**Figure 1:**
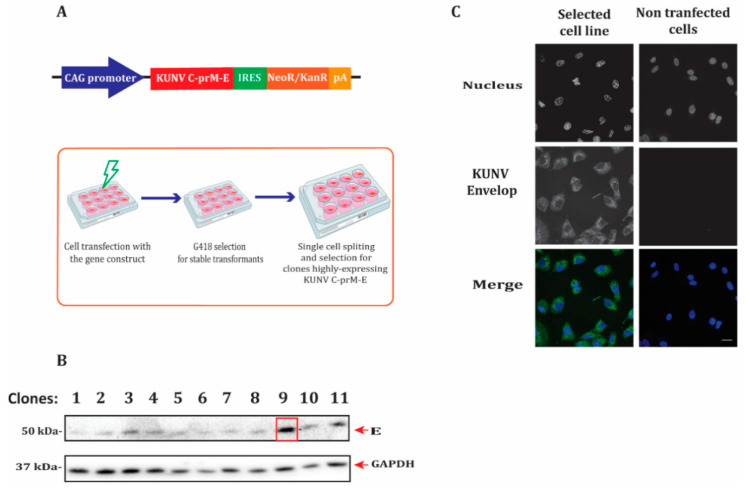
Establishment of a stable cell line expressing the Kunjin virus (KUNV) packaging system of capsid (C), precursor membrane (prM), and envelope (E), C-prM-E. (**A**) Schematic illustration of the gene construct expressing C-prM-E having G418 antibiotic resistance. Gene expression is driven by the CAG promoter. The sequence coding C-prM-E was fused with an internal ribosome entry site (IRES) sequence and the neomycin/kanamycin resistance (NeoR/KanR) gene, followed by the polyadenylation signal (pA). To generate baby hamster kidney cells (BHK)-21 stably expressing KUNV C-prM-E, the gene construct was transfected into BHK-21 cells, followed by selection for transfected cells with G418. Cells were then separated into single cells and grown as individual clones. The schema was generated by using the Biorender web tool. (**B**) Immunoblotting of the cell lysates of each clone (1–11) to examine the expression level of E protein. The expression levels were normalized using the endogenous Glyceraldehyde-3-phosphate dehydrogenase (GAPDH) protein as control. (**C**) Immunofluorescence staining of cells from clone 9 with the E antibody. The nucleus was counterstained by DAPI (blue). The bar scales represent 20 µm.

To generate a system producing high levels of RVPs, we initially established a BHK-21 stable cell line expressing high levels of KUNV structural proteins. The KUNV C-prM-E-IRES-NeoR/KanR construct (Figure 1A) was transfected into BHK-21 cells and selected for cells having G418 resistance. Transformants were separated into single cells and seeded into multiwell plates (Figure 1A). These cells were grown as individual clones, and protein expression was examined by immunoblotting (Figure 1B). Following production, the KUNV polyprotein is cleaved into individual structural proteins by host proteases [27]; thus, the E protein functions as a suitable marker for confirming gene expression and was detected by immunoblotting. We found that cell clone 9 had high levels of E protein compared to all the other clones (Figure 1B). Furthermore, immunofluorescent labeling of clone 9 cells using an E protein antibody indicated that E protein was expressed in all cells (Figure 1C). Cell clone 9 was amplified further and selected as our RVP packaging cell line for further use. 

### 3.2. Establishment of an Enhanced System for the KUNV Reporter Virus-Like Particles Production 

**Figure 2:**
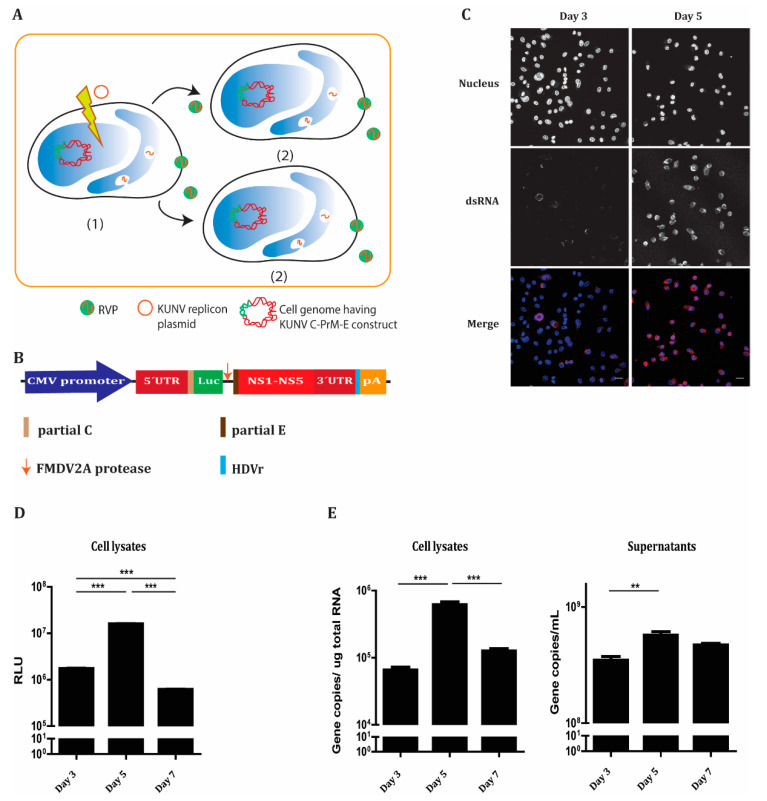
Construction of an enhanced KUNV reporter virus-like particle (RVP) production system. (**A**) The scheme illustrates the infectious cycles to form RVPs. (1) The DNA KUNV replicon is transfected into the stable cell line expressing C-prM-E. The RNA replicon is then expressed and replicated inside replication compartments. The RNA replicon is packaged by C, prM, and E, forming RVPs (2). The RVP progeny may infect and replicate in neighboring C-prM-E expressing cells, forming additional RVPs. (**B**) Schematic illustration of the DNA-based KUNV replicon construct. The replicon is driven by the Cytomegalovirus (CMV) promoter expressing an open reading frame flanked by the 5′- untranslated region (UTR) and the 3′-UTR comprising: first, 81 nucleotides of the C gene fused in frame with the firefly luciferase gene (Luc) as a reporter gene, the foot-and-mouth disease virus autoprotease 2a (FMDV 2A) and, last, 84 nucleotides of the E gene and all the nonstructural proteins. The antigenomic hepatitis delta virus ribozyme (HDVr) sequence was inserted immediately downstream of the KUNV 3′-UTR, followed by the Simian virus 40 (SV40) polyadenylation signal (pA). (**C**) Immunofluorescence labeling of the C-prM-E stable cells transfected with the replicon construct 3 days and 5 days post-transfection with an antibody against dsRNA (red). The nucleus was counterstained by DAPI (blue). Bar scales represent 20 µm. (**D**) Relative luciferase units (RLU) from cell lysates 3–7 days post-transfection. (**E**) Gene copy numbers of replicon from cell lysates, and cell culture supernatants 3–7 days post-transfection as measured by qPCR. The experiments were conducted independently three times with two technical repeats. The *p* values are indicated using ** *p* < 0.01 and *** *p* < 0.001.

To enhance KUNV RVP production, the cells expressing C-prM-E were used as a system to supply structural proteins (Figure 2A) together with the DNA-based KUNV replicon (Figure 2B). Within cells, the expressed replicon RNA was packaged by structural proteins, generating RVPs. The J2 antibody was used to stain double-stranded (ds) RNA, which could be visualized in the endoplasmic reticulum (ER) membrane at day 3 post-transfection, indicated that approximately 10% of the cells were transfected with the DNA replicon (Figure 2C). On day 5, dsRNA became abundant in most cells (Figure 2C), which showed that the expressed RNA replicons were capable of replicating and had been transferred to neighboring cells via the packaging machinery. The expression of the replicon was monitored by the luciferase activity that showed an increase over time (Figure 2D). The maximum relative luminescence units (RLU) were detected 5 days post-transfection, which indicated a suitable time point for successful RVP production (Figure 2D). Consistently, we observed a similar trend in the replicon gene copy number as measured by qPCR. The gene copy numbers reached the maximum level in both cell lysates and supernatants 5 days post-transfection before declining at day 7 (Figure 2E). Our data demonstrate the ability of KUNV RVPs to spread from transfected cells to neighboring cells; thus, our C-prM-E stable cell line was shown to be a good system to enhance KUNV RVP production.

**Figure 3:**
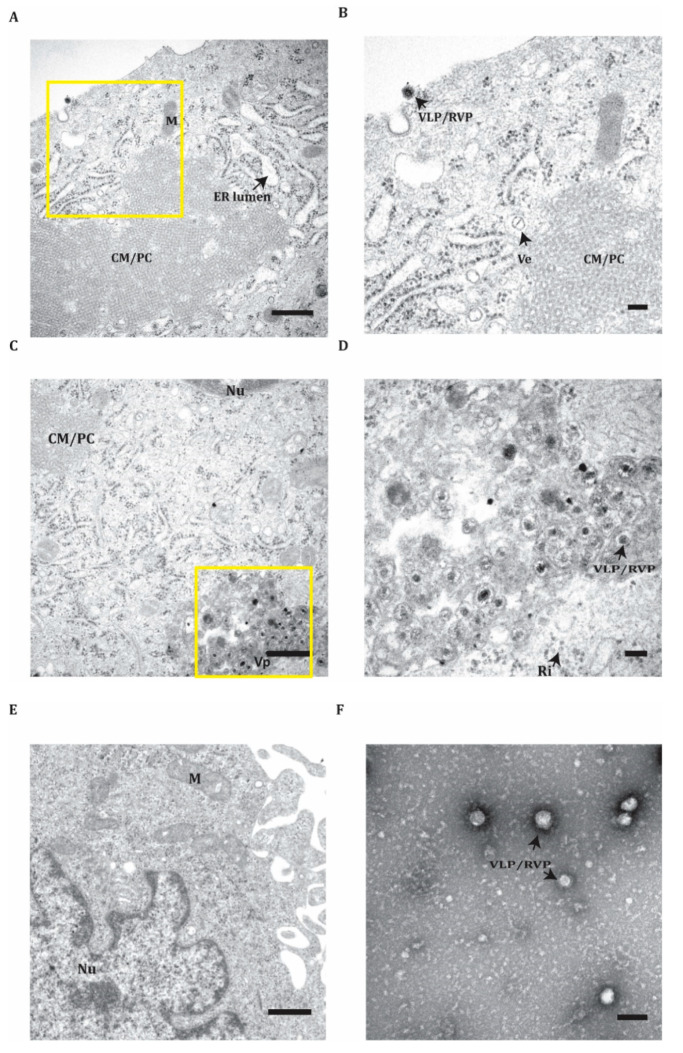
Transmission electron microscopy images of the RVP production system. (**A**–**D**) Images representing the C-prM-E stable BHK-21 cell line transfected with the KUNV replicon construct. (**A**,**C**) represent different subcellular areas, whereas (**B**,**D**) show higher magnification images of the yellow boxes indicated in the (**A**) and the (**C**), respectively. (**E**) The image represents BHK-21 cells used as control. (**F**) The image of cell culture supernatants from the C-prM-E stable cells transfected with the KUNV replicon. ER: the endoplasmic reticulum; Nu: nucleus; M: mitochondria; CM/PC: convoluted membranes, paracrystalline structure; Vp: vesicle packets; Ve: virus-induced vesicles in the ER; VLP/RVP: virus-like particle, reporter virus-like particles; Ri: ribosome. Scale bars represent 500 nm in (**A**,**C**,**E**) and 100 nm in (**B**,**D**,**F**).

To further verify the RVP production system, we analyzed the C-prM-E cells transfected with the DNA replicons using TEM. This revealed RVPs together with structures commonly found during KUNV replication [28,29,30]. At the cellular surface, we detected particles leaving the cells via exocytosis (Figure 3A,B). We also observed the convoluted membranes/paracrystalline structures and vesicle packets (VPs) structures (Figure 3A,B). In close proximity to the nucleus, we detected an abundance of VLP/RVPs inside VPs (Figure 3C,D). These structures were not detected in the BHK-21 control cells (Figure 3E). In addition, we detected VLP/RVPs in the supernatants of the RVP system cells (Figure 3F).

### 3.3. KUNV RVPs Are Only Infectious for a Single Round

**Figure 4:**
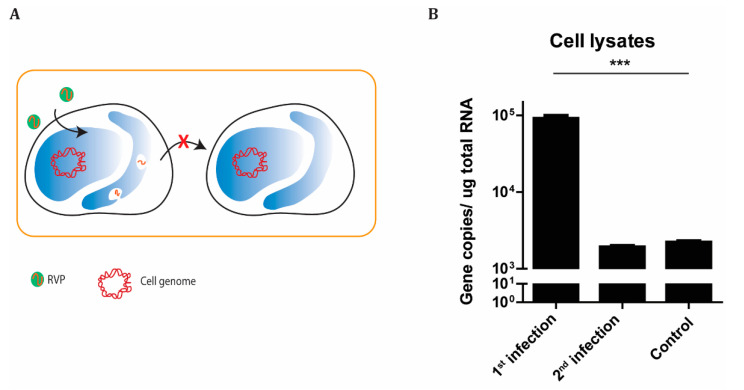
KUNV RVPs infect cells in a single round. (**A**) Schematic illustration of the process of RVP infection of BHK-21 cells. Hereby, the absence of a packaging system in the infected cells limits additional RVP production. (**B**) Gene copy numbers of the KUNV replicon in cell lysates after first- and second-round infection and uninfected control lysates as measured by qPCR. The experiments were conducted independently three times with two technical repeats. The *p* values are indicated using *** *p* < 0.001.

As putative recombinations between the DNA replicon and genes of the packaging system could produce an active KUNV, we tested the formed RVPs using continuous infection cycles. Cell culture supernatant from the RVP production system was used to infect BHK-21 cells for the first round of infection. Supernatant from these cells was then used to infect another batch of BHK-21 cells in the second round (Figure 4A). After the first-round infection, the replicon copy number was significantly higher than the uninfected control lysates (Figure 4B). On the contrary, no viral RNA was detected in the second round when compared to the control (Figure 4B). There was a background level of gene copy numbers in the control and the second round-infecting lysates, while the replicon copy number was undetectable in the blank. Thus, this background level may result from the non-specificity of primers during cDNA generation. Altogether, these results show that RVPs only infect the cells for a single cycle and that no recombination resulting in the production of an active virus had occurred.

### 3.4. EBOV GP or EBOV VP40 Gene Transduction by KUNV RVPs

**Figure 5:**
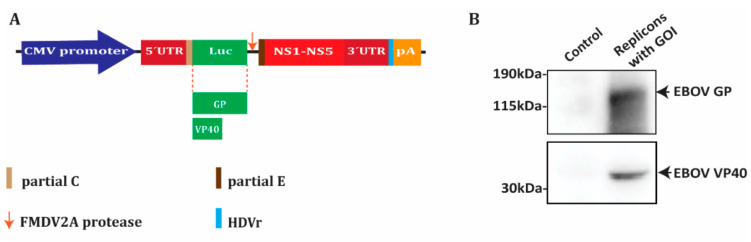
Expression of EBOV vaccine candidates using the KUNV replicon. (**A**) The luciferase reporter gene (Luc) was substituted with genes coding EBOV GP or VP40 in the DNA replicon. (**B**) Immunoblotting of cell lysates 2 days after transfection with EBOV GP or EBOV VP40 replicons versus the untransfected cell control.

As KUNV RVPs only infect cells in a single round, they may safely be used for the delivery of putative vaccine candidate genes after in vivo immunization. As both EBOV GP and VP40 were predicted to be potential antigenic targets to develop a novel Ebola vaccine, we decided to test their expression in the KUNV DNA replicon system. We substituted the luciferase reporter gene with EBOV GP or VP40, respectively (Figure 5A). By immunoblotting, we showed that EBOV GP was expressed at the expected size of around 150 kDA (Figure 5B). We also detected a band at 40 kDa using a specific antibody against EBOV VP40 (Figure 5B). 

**Figure 6:**
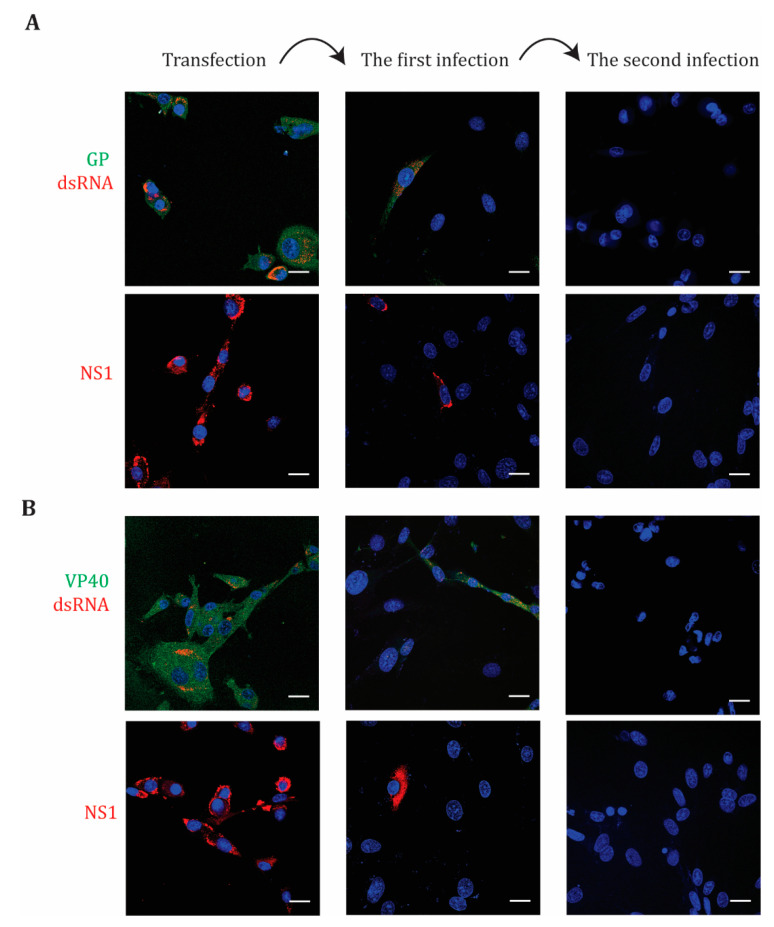
Immunofluorescence labeling of BHK-21 C-prM-E cells after transfection with the KUNV replicons expressing EBOV GP or VP40 proteins (**A**,**B**), respectively, followed by two cycles of RVP infections of BHK-21 cells. The cells were visualized with the antibodies anti-EBOV GP (green), EBOV VP40 (green) (**A**,**B**), respectively, and the antibodies anti-dsRNA (red) and KUNV NS1 (red). The nucleus was counterstained with DAPI (blue). Bar scales represent 20 µm.

To examine the transduction of EBOV GP or VP40 by RVPs prior to animal studies, the cells were labeled with specific antibodies after replicon-transfection and after the first and the second rounds of RVP infection (Figure 6). We showed that the replicons expressed EBOV GP or VP40 in the transfected cells (Figure 6A,B). As expected, the replicon RNA was delivered by RVPs into BHK-21 cells only in the first round of cell infection. These results verify that RVPs containing EBOV GP or VP40 were produced and that no recombination occurred within the system. The detection of NS1 expression verifies the production of the polyprotein, while the presence of dsRNA indicates the flavivirus-like replication (Figure 6A,B). 

### 3.5. RVPs Induced Seroconversion 

**Figure 7:**
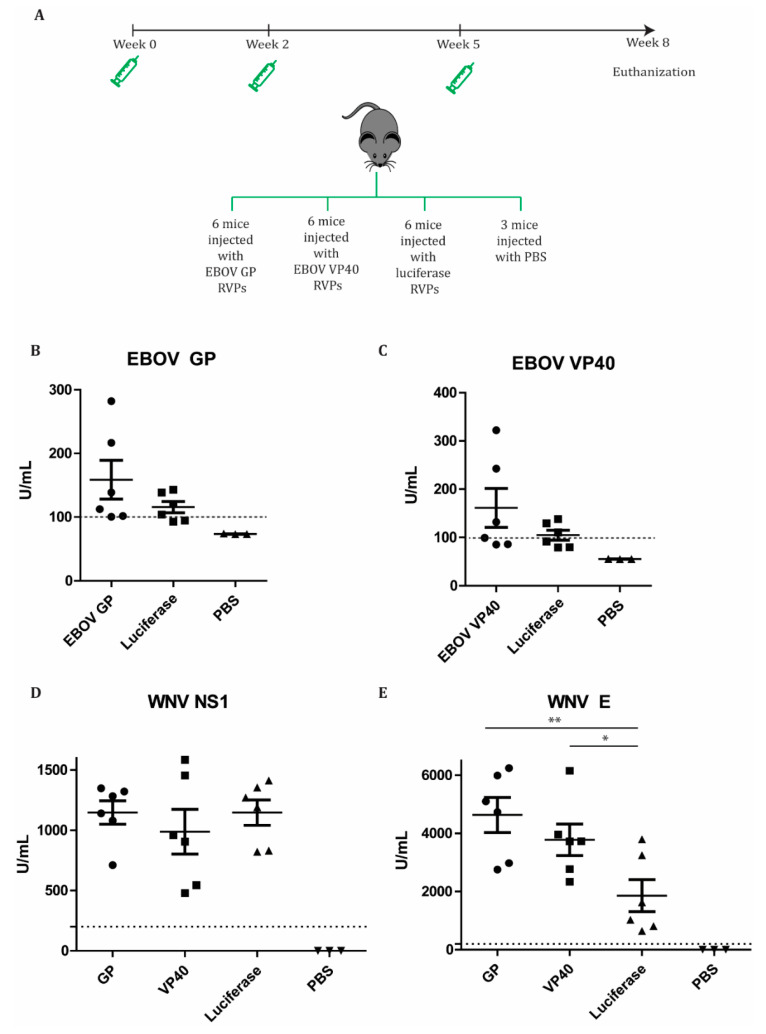
RVPs induced seroconversion. (**A**) Schematic illustration of the mice immunization schedule. Mice sera from the study groups were assayed with enzyme-linked immunosorbent assays (ELISA) to measure antibodies against EBOV GP (**B**), EBOV VP40 (**C**), WNV NS1 (**D**), and WNV E (**E**). The dashed line indicates the detection limit as suggested by the kit manufacturers. The assays were conducted with two technical repeats. The *p* values are indicated using * *p* < 0.05 and ** *p* < 0.01.

As we successfully demonstrated that our RVPs could transduce cells, we wanted to examine their in vivo immunogenicity by administering them into mice (Figure 7A). During the study, all mice showed no difference in health status and weights compared to control groups (Table S1). In the group of EBOV GP RVP-immunized mice, four out of six mice had antibodies against the protein above the detection threshold (100 U/mL) suggested by the kit´s manufacturer (Figure 7B). Consistently, three out of the six mice immunized with EBOV VP40 RVPs had antibody levels above the threshold (Figure 7C). There were two mice in the GP and the VP40 immunized groups, respectively that had antibody levels higher than all mice in the control groups (Figure 7B,C). In addition, the immunogenicity of these RVPs was further tested as a putative WNV vaccine candidate. The mice injected with luciferase RVPs showed significantly high amounts of antibodies for WNV NS1 and the E protein compared to the PBS-injected mice (Figure 7D,E). EBOV GP and VP40 transductions by RVPs did not compromise WNV NS1 and E seroconversion (Figure 7D,E). Interestingly, the mice immunized with GP and VP40 RVPs showed significantly higher titers of WNV E antibodies compared to the mice immunized with luciferase-expressing RVPs (Figure 7E). In short, these results suggest that the RVPs successfully delivered the replicon to mouse cells. The replicons expressed the target genes, EBOV GP, and VP40, which elicited seroconversion in mice (Figure 7B,C). In addition, KUNV NS1 expressed by the replicons and KUNV E delivered as RVP surface protein provoked WNV seroconversion in mice (Figure 7D,E).

## 4. Discussion

The KUNV RVPs are engineered particles that contain replicons having no viral packaging genes. This property restricts their infectability to a single round; thus, they cannot cause systemic infection. As a strategy for vaccine development, they are a compromise between inactivated and live-attenuated viruses. Unlike inactivated virus particles, RVPs can infect and transduce antigen-presenting cells like macrophages or dendritic cells to express vaccine candidate proteins and, therefore, elicit stronger immune responses. Compared to live-attenuated virus vaccines, RVPs infect cells only in a single round, which makes them a safer vaccine platform. Furthermore, KUNV is an attenuated subtype of WNV whose clinical symptoms are less detrimental than those of WNV [2]. In addition, KUNV, like other flaviviruses, does not integrate its genome into that of the host cell and does not induce abnormal cell development [31]. Therefore, KUNV RVPs could be a safe vaccine system suitable for in vivo administration. 

To enhance the production of RVPs, we generated a stable cell line expressing KUNV C-prM-E as a packaging system for the replicons. We transfected BHK-21 cells with the construct KUNV C-prM-E-IRES-NeoR/KanR and selected for transformants with high expression of C-prM-E. In our view, this strategy to establish the stable cell line is safer than that using a lentivirus system to integrate the KUNV packaging system genes in cells, or using the recombinant Semliki Forest virus (SFV) RNA replicon expressing KUNV structural proteins in trans as previously described [32]. Our approach of making the stable cell line eliminated the risk that the unexpected recombinations of lentivirus or SFV genes with KUNV packaging genes could lead to the formation of chimeric progenies. Contamination of KUNV RVPs with such potential chimeric particles would undermine the concept of single-round-infecting RVPs being safer for vaccine development.

In this study, we characterized the potential and the safety of using KUNV RVPs to deliver other genes of interest as a strategy for developing vaccines against other viruses concomitantly with WNV. We generated KUNV RVPs that deliver genes coding the EBOV envelope GP and the matrix VP40 protein into cells. Without the EBOV capsid proteins, these two proteins cannot function as an EBOV packaging system. Thus, there is no risk for the development of a chimeric KUNV/EBOV virus using the RVP production system. Indeed, we showed that the RVPs having these genes only infected cells in a single round, similarly to the luciferase RVP (Figure 4B and Figure 6). Mice injected with these RVPs survived and showed no differences in weights and health status, compared to the two control groups of luciferase RVPs and PBS (Table S1). Immunizations of these RVPs elicited seroconversions to these antibodies in mice. KUNV RVPs also conferred the production of antibodies against the NS1 and E proteins of WNV. These results were expected due to the close relationship between KUNV and WNV, given that KUNV is an attenuated strain of WNV [2,3]. Furthermore, the expression of EBOV GP or VP40 did not attenuate the production of antibodies against the WNV NS1 and E proteins. Instead, the EBOV proteins seem to have a significant adjuvating effect on the immunogenicity of the WNV E protein (Figure 7E), which requires further investigation.

There are four approved EBOV vaccines in use currently: a recombinant adenovirus-based vector vaccine prior to a boost with a vaccinia-based vector vaccine (Ad26.ZEBOV/MVA-BN), a single dose Ad5.ZEBOV vaccine, a recombinant Vesicular stomatitis virus-based vector vaccine (rVSV-EBOV), and a dose of an rVSV vaccine followed by a boost with an Ad5 vaccine [33]. However, only the rVSV-EBOV produced by Merck is prequalified by the World Health Organization [34]. In addition, several other vaccine systems have shown high protection against EBOV in non-human primates (NHP) [20]. Among them, the EBOV VLP platform is a safe approach due to its lack of the viral genome. However, since the VLPs are produced by transient expression of VP40 alone or with GP in mammalian cells and they cannot replicate to generate progeny particles, the approach is costly to scale up [35]. Similarly to the KUNV RVP strategy, however, the Venezuelan equine encephalitis virus-based RVP platforms have shown inconsistent results [36,37]. On the other hand, immunizations with KUNV RVPs delivering EBOV GP could result in 80% protection in guinea pigs and NHP in previous studies [16,38]. In this study, we explored the use of KUNV RVPs delivering EBOV VP40 gene in comparison with EBOV GP, and we demonstrated that these candidates provoked immunogenicity in mice.

To our knowledge, this is the first time that KUNV RVPs delivering EBOV VP40 to elicit immune response has been shown and their levels of seroconversion were similar to GP RVPs. It has previously been shown that the expression of EBOV VP40 in the absence of GP can generate VLPs [22,39,40], which might be due to the ability of VP40 to bind host proteins [41,42]. Several studies have suggested that VP40 has the potential to be immunogenic [24,25,26]. The protein can also stimulate natural killer (NK) cells to protect studied mice from EBOV [23]. Thus, it is interesting to study the NK cell response during the administration of VP40 RVPs. In addition to the individual RVP immunization and challenging experiment, we intend to combine the expression of GP/VP40 RVPs. This will determine if any enhanced protection effect can be reached as co-expression of GP EBOV protein can enhance VP40 release approximately by 40-fold [39].

In addition, the titers of seroconversion to EBOV GP and VP40 by RVPs from our platform were not as high as in a previous study [38]. This might be due to a lower dose of RVPs that was administrated (approximately 10^6^ infectious RVPs) per mouse. In addition to dosages of RVPs, our results can be improved by evaluating the antibody titers over the course of immunization which could progressively increase prior to animal euthanization as showed by Melen et al. [25].

Furthermore, the overexpression of GP protein can trigger cytopathic effects in culturing cells including cell rounding and detachment from the extracellular matrix [43]. The induced morphological changes were due to the downregulation of cellular surface proteins involved in cellular adhesion [44]. However, we did not observe these severe cytopathic effects during the expression of EBOV GP and gene transduction by RVPs (data not shown). Mice injected with EBOV GP appeared to be healthy, showing no changes in weight (Table S1) when compared to control mice. Thus, we speculate that our KUNV RVP system did not trigger strong cytopathic effects. This is consistent with a previous finding showing that constitutive, moderate EBOV GP expression does not elicit cytotoxicity [45].

## 5. Conclusions

In conclusion, we developed a safe platform to enhance KUNV RVP production through the combination of the stable cell line expressing C-prM-E and DNA replicon transfection. KUNV RVPs were able to transduce cells to express EBOV vaccine candidate genes GP and VP40 in a single round. The RVPs elicited immune responses to EBOV GP, EBOV VP40, WNV NS1, and WNV E proteins. In future, the efficacy of our RVP system as a vaccine strategy will be analyzed further by challenging study animals with viruses. 

## Supplementary Materials

The following are available online at https://www.mdpi.com/2076-2607/8/12/1890/s1, Table S1: Animal weights during immunization.
