# Supplementary material for: Development of a Multivalent Kunjin Virus Reporter Virus-Like Particle System Inducing Seroconversion for Ebola and West Nile Virus Proteins in Mice
Source: Microorganisms. 2020 Nov 29;8(12):1890. doi: 10.3390/microorganisms8121890 (PMC7760487; doi:10.3390/microorganisms8121890)
Supplement: Supplementary file 1 [file microorganisms-08-01890-s001.pdf]

microorganisms

Supplementary Materials

Table 1. Average weights of mice from EBOV VP40 RVPs, EBOV GP RVPs, Luciferase RVPs, or PBS immunized groups beginning from one week before the first immunization (week −1) to the euthanized date (week 8). N is the number of mice.

**Week**	EBOV VP40 RVPs			EBOV GP RVPs			Luciferase RVPs			PBS		
	Mean	SEM	N	Mean	SEM	N	Mean	SEM	N	Mean	SEM	N
Week −1	15.55	0.58	6	15.25	0.43	6	15.02	0.48	6	15.6	0.7	3
Week 0	17.62	0.63	6	17.2	0.39	6	16.98	0.39	6	17.57	0.43	3
Week 1	17.85	0.58	6	17.61	0.3	6	17.53	0.31	6	18,00	0.31	3
Week 2	18.72	0.58	6	18.48	0.25	6	18.07	0.38	6	18.53	0.26	3
Week 3	19.07	0.61	6	19.18	0.24	6	18.82	0.3	6	19.27	0.37	3
Week 4	19.43	0.7	6	19.73	0.3	6	19.08	0.29	6	19.57	0.44	3
Week 5	19.88	0.73	6	19.97	0.28	6	19.28	0.3	6	20.03	0.42	3
Week 6	20.12	0.75	6	20.38	0.24	6	19.72	0.37	6	20.27	0.44	3
Week 7	20.47	0.74	6	20.28	0.33	6	20.07	0.35	6	20.47	0.56	3
Week 8	21.13	0.7	6	20.95	0.55	6	20.82	0.42	6	21.17	0.41	3

Microorganisms **2020**, *8*, 1890; doi:10.3390/microorganisms8121890 www.mdpi.com/journal/microorganisms
